# Variation in cartilage T2 and T2* mapping of the wrist: a comparison between 3- and 7-T MRI

**DOI:** 10.1186/s41747-023-00394-1

**Published:** 2023-12-14

**Authors:** Rafael Heiss, Marc-André Weber, Eva L. Balbach, Maximilian Hinsen, Frederik Geissler, Armin M. Nagel, Mark E. Ladd, Andreas Arkudas, Raymund E. Horch, Christine Gall, Michael Uder, Frank W. Roemer

**Affiliations:** 1 Department of Radiology, University Hospital Erlangen, Friedrich-Alexander-Universität Erlangen-Nürnberg (FAU), Maximiliansplatz 3, 91054 Erlangen, Germany; 2 Institute of Diagnostic and Interventional Radiology, Pediatric Radiology and Neuroradiology, University Medical Center Rostock, Schillingallee 35, 18057 Rostock, Germany; 3 Medical Physics in Radiology, German Cancer Research Center (DKFZ), Im Neuenheimer Feld 280, 69120 Heidelberg, Germany; 4 Faculty of Medicine and Faculty of Physics and Astronomy, Heidelberg University, Im Neuenheimer Feld 226, 69120 Heidelberg, Germany; 5 Department of Plastic and Hand Surgery and Laboratory for Tissue Engineering and Regenerative Medicine, University Hospital Erlangen, Friedrich-Alexander-Universität Erlangen-Nürnberg (FAU), Krankenhausstraße 12, 91054 Erlangen, Germany; 6 Institute for Medical Informatics, Biometry and Epidemiology, Friedrich-Alexander-Universität Erlangen-Nürnberg (FAU), Waldstraße 6, 91054 Erlangen, Germany; 7 Boston University School of Medicine, 72 E Concord St, Boston, MA 02118 USA

**Keywords:** Cartilage (articular), Image processing (computer-assisted), Magnetic resonance imaging, Triangular fibrocartilage, Wrist joint

## Abstract

**Background:**

To analyze regional variations in T2 and T2* relaxation times in wrist joint cartilage and the triangular fibrocartilage complex (TFCC) at 3 and 7 T and to compare values between field strengths.

**Methods:**

Twenty-five healthy controls and 25 patients with chronic wrist pain were examined at 3 and 7 T on the same day using T2- and T2*-weighted sequences. Six different regions of interest (ROIs) were evaluated for cartilage and 3 ROIs were evaluated at the TFCC based on manual segmentation. Paired *t*-tests were used to compare T2 and T2* values between field strengths and between different ROIs. Spearman’s rank correlation was calculated to assess correlations between T2 and T2* time values at 3 and 7 T.

**Results:**

T2 and T2* time values of the cartilage differed significantly between 3 and 7 T for all ROIs (*p* ≤ 0.045), with one exception: at the distal lunate, no significant differences in T2 values were observed between field strengths. T2* values differed significantly between 3 and 7 T for all ROIs of the TFCC (*p* ≤ 0.001). Spearman’s rank correlation between 3 and 7 T ranged from 0.03 to 0.62 for T2 values and from 0.01 to 0.48 for T2* values. T2 and T2* values for cartilage varied across anatomic locations in healthy controls at both 3 and 7 T.

**Conclusion:**

Quantitative results of T2 and T2* mapping at the wrist differ between field strengths, with poor correlation between 3 and 7 T. Local variations in cartilage T2 and T2* values are observed in healthy individuals.

**Relevance statement:**

T2 and T2* mapping are feasible for compositional imaging of the TFCC and the cartilage at the wrist at both 3 and 7 T, but the clinical interpretation remains challenging due to differences between field strengths and variations between anatomic locations.

**Key points:**

•Field strength and anatomic locations influence T2 and T2* values at the wrist.

•T2 and T2* values have a poor correlation between 3 and 7 T.

•Local reference values are needed for each anatomic location for reliable interpretation.

**Graphical Abstract:**

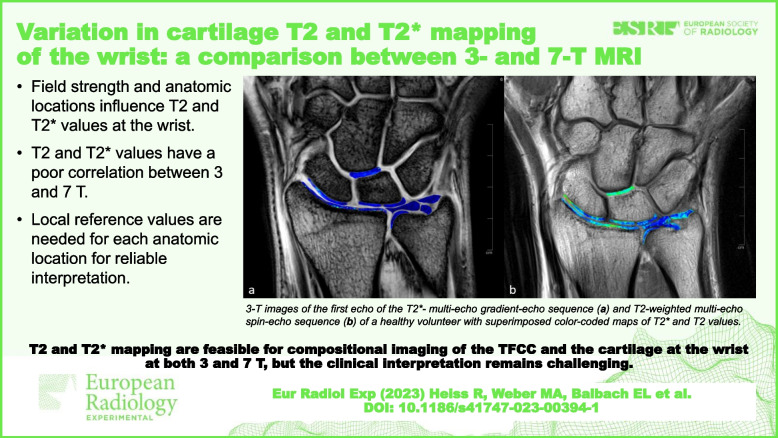

**Supplementary Information:**

The online version contains supplementary material available at 10.1186/s41747-023-00394-1.

## Background

The wrist is one of the most challenging anatomic areas to examine by magnetic resonance imaging (MRI) due to the small size of tissues including the thin articular cartilage at multiple locations and the triangular fibrocartilage complex (TFCC) with its complex anatomy [1]. Detection of early, premorphologic alterations of the cartilage or the TFCC may be clinically relevant in a presurgical assessment prior to reconstructive wrist surgery and may also have therapeutic implications in the sports context [2–4].

Conventional MRI usually depicts morphologic degenerative changes at the cartilage and the TFCC only at advanced stages of disease [1]. In contrast, compositional MRI promises to overcome these issues by depicting biochemical tissue properties much earlier than morphologic changes can be detected [5, 6]. T2 and T2* relaxometry (“mapping”) are two widely applied techniques that enable non-invasive quantification of tissues’ water content and assessment of collagen content and organization [7, 8]. Higher magnetic field strengths with higher contrast-to-noise and signal-to-noise ratios allow for high spatial resolution imaging allowing the application of compositional MRI techniques also for small anatomical structures not achievable with lower field strengths [9].

However, at 3 T, T2 mapping data for the cartilage or TFCC at the wrist are limited to a few feasibility studies [1, 10, 11]. Moreover, to date, systematic tissue assessment applying T2 and T2* mapping at the wrist at 7 T is unavailable. Despite the potential advantages of 7-T MRI of the wrist, studies comparing morphologic 7-T and 3-T MRI have not shown unequivocal superiority of the former for assessment of different joint tissues [12–15]. This is related to the lack of dedicated radiofrequency coils and to the technical challenges of ultra-high-field MRI, including inhomogeneity in the transmit field (B_1_^+^) distribution and chemical shift artifacts, which may also affect compositional imaging such as T2 and T2* mapping [14, 16]. In addition, regional variability in T2 and T2* values for healthy tissue at different anatomic locations, which has been reported for the articular cartilage of the knee and hip, has not been described for compositional imaging at the wrist [17, 18]. Knowledge of local variations in compositional imaging is necessary for valid image interpretation based on quantification.

We hypothesized that T2 and T2* values differ between field strengths, but show excellent correlation, and that T2 and T2* time values show significant variability between different anatomic locations in healthy articular cartilage and in different regions of interest (ROIs) of the TFCC at the same field strength. Hence, the aims of this study were (1) to compare T2 and T2* values obtained at 3 and 7 T for articular cartilage and the TFCC at different anatomic locations in healthy controls and patients with chronic wrist pain; (2) to assess the correlation of T2 and T2* values between field strengths; and (3) to compare T2 and T2* values of articular cartilage and the TFCC in healthy controls at 3 and 7 T to determine whether these values vary with anatomic location.

## Methods

### Study sample

**Fig. 1:**
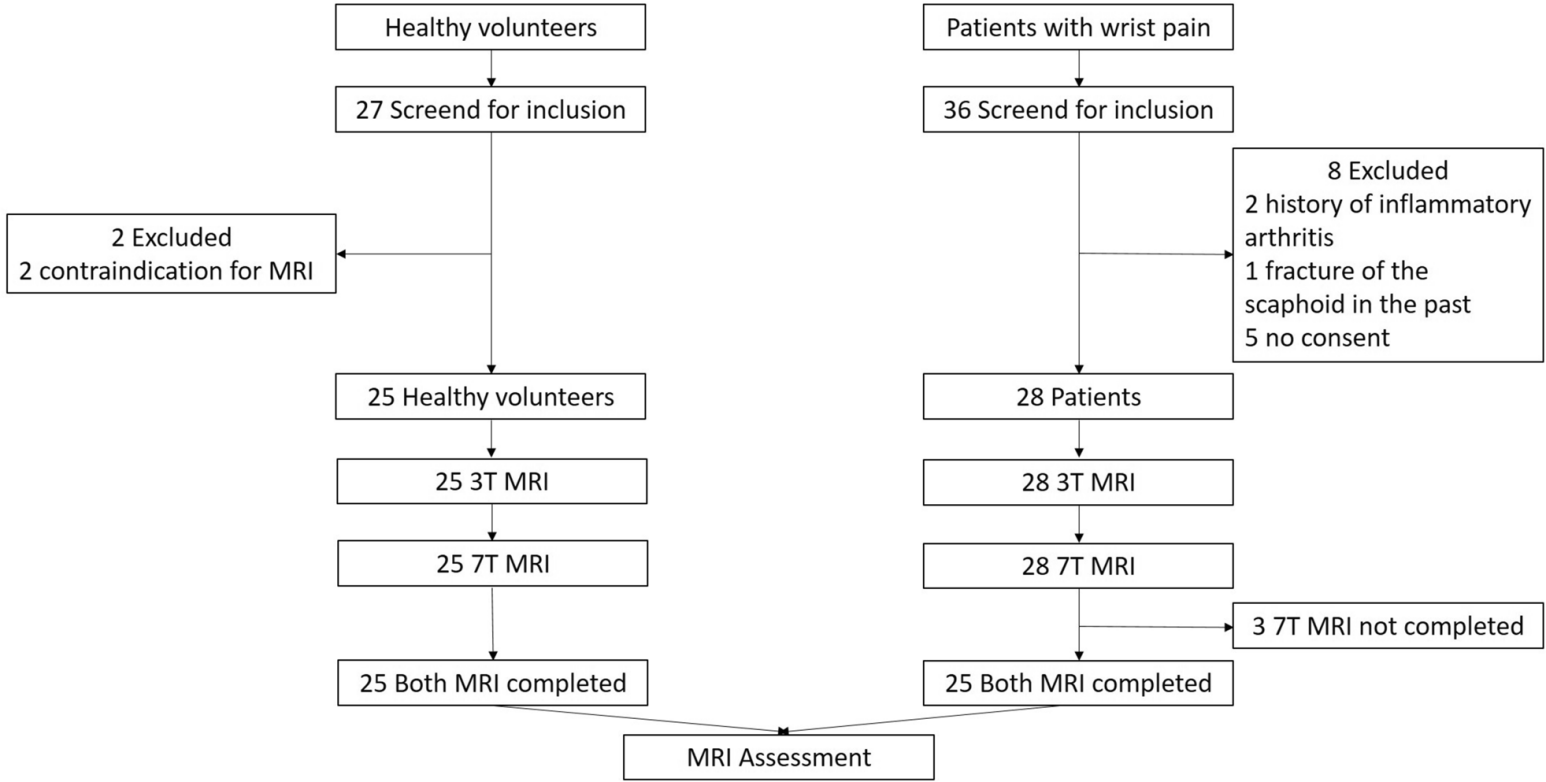
Flowchart of the study

Participants were prospectively enrolled between July 2018 and June 2019 after providing written informed consent. The study sample consists of 25 patients and 25 healthy volunteers (Fig. 1). Patients with chronic wrist pain (of more than three months) who were referred for outpatient consultation at a tertiary referral center for hand surgery were asked to participate in the study. The exclusion criteria were a history of trauma within the last six months, suspicion of carpal tunnel syndrome, any previous surgical intervention at the wrist, any past fracture of the distal forearm or carpal bones, a history of inflammatory arthritis, and inability to undergo 3-T or 7-T MRI.

Results based on multi-tissue ordinal expert assessment comparing 3-T and 7-T results in the same study cohort have recently been published [12].

### MRI protocol

All study participants were examined at 3 T (Magnetom Vida, Siemens Healthineers, Erlangen, Germany) and 7 T (MAGNETOM Terra, Siemens Healthineers) with dedicated wrist coils on the same day. All participants underwent 3-T MRI first. For all measurements at 3 T, a 16-channel receive hand–wrist radiofrequency coil was used. For excitation, the integrated body coil was used. For 7-T MRI, a 1-channel transmit/16-channel receive wrist radiofrequency coil (Rapid Biomedical GmbH, Rimpar, Germany) was employed. The transmit part consists of a separate quadrature birdcage coil. At 7 T, a coronal multi-echo spin-echo T2-weighted sequence (repetition time [TR] 2,000 ms; echo time [TE] 16.1 ms, 32.2 ms, 48.3 ms, 64.4 ms, and 80.5 ms; acquisition time 06:52 min:s; voxel size 0.3 × 0.3 × 2.0 mm; flip angle 180°; bandwidth 434 Hz) and multi-echo gradient-echo T2*-weighted sequence (TR 648 ms; TE 4.08 ms, 7.01 ms, 9.62 ms, 12.23 ms, and 15.29 ms; acquisition time 04:10 min:s; voxel size 0.3 × 0.3 × 3.0 mm; flip angle 60°; bandwidth 470 Hz) were acquired.

At 3 T, protocols with image acquisition times comparable to those used at 7 T were applied: a coronal multi-echo spin-echo T2-weighted sequence (TR 2,000 ms; TE 16.1 ms, 32.2 ms, 48.3 ms, 64.4 ms, and 80.5 ms; acquisition time 06:52 min:s; voxel size 0.3 × 0.3 × 3.0 mm; flip angle 180°; bandwidth 228 Hz) and a coronal multi-echo gradient-echo T2*-weighted sequence (TR 648 ms; TE 6.04 ms, 16.94 ms, 27.84 ms, 38.74 ms, and 49.64 ms; acquisition time 04:10; voxel size 0.3 × 0.3 × 3.0 mm; flip angle 60°; bandwidth 260 Hz). Detailed MRI parameters are provided in Supplementary Table S1. The scan time including coronal T1-weighted turbo spin-echo, coronal fat-suppressed proton-density-weighted turbo spin-echo, and transversal T2-weighted turbo spin-echo images (not part of this evaluation) amounts to a total acquisition time of 22:35 min:s at 7 T and 23:48 min:s at 3 T. Both T2 and T2* maps were generated by the vendor’s standard software *syngo* MapIt (Siemens Healthineers) [1]. T2 and T2* relaxation times were derived from T2/T2* parameter maps using a pixel-wise, monoexponential least-squares-fit analysis [1].

### MRI assessment

**Fig. 2:**
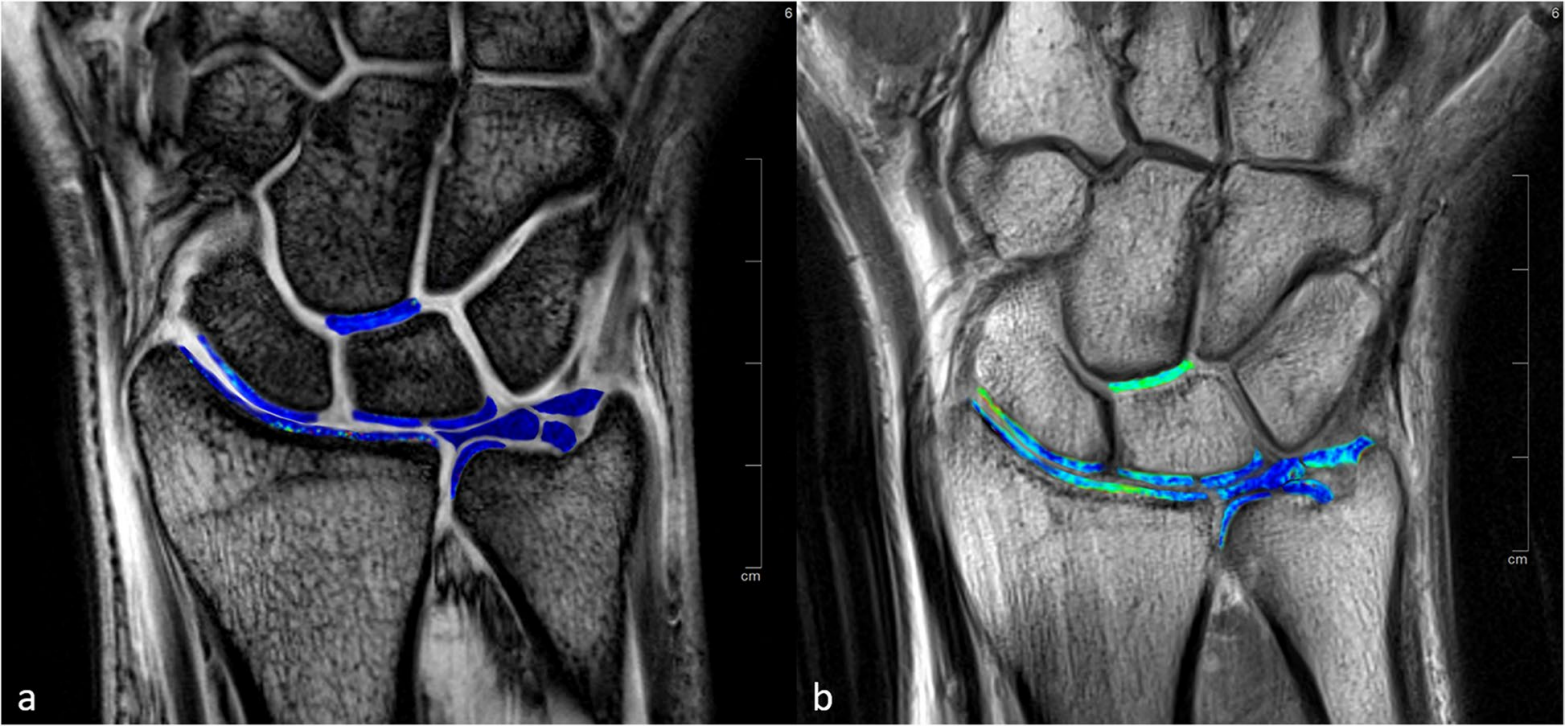
Three-Tesla images of the first echo of the T2*-weighted multi-echo gradient-echo sequence (**a**) and T2-weighted multi-echo spin-echo sequence (**b**) of a healthy volunteer with superimposed color-coded maps of T2* and T2 values. Regions of interest (ROIs) were placed in the cartilage of the distal radius, in the proximal scaphoid, in the proximal radial and ulnar lunate, in the distal radioulnar joint, and in between the distal lunate and proximal capitate. Three ROIs were placed at the triangular fibrocartilage complex: at the central disk and at both the foveal and apical attachments. The same analysis was performed for 7-T MRI

All images were blinded for the whole assessment and were evaluated by a radiologist (R.H.) with eight years of experience in musculoskeletal MRI and a special interest in wrist imaging. T2 and T2* maps were assessed by placing ROIs at nine anatomic locations. Six of these ROIs were placed in the cartilage of the distal radius, in the proximal scaphoid, in the proximal radial lunate and ulnar lunate, in the distal radioulnar joint, and in between the distal lunate and proximal capitate [1]. Three ROIs were placed at the TFCC [19]: at the central disk and at both the foveal and the apical attachment of the TFCC (Fig. 2). The images were magnified on a workstation monitor to enable optimal visualization of the anatomy and to optimize ROI placement. ROIs were manually placed in each anatomic location using the images of the first echoes of the T2 multi-echo spin-echo and T2* multi-echo gradient-echo sequences by omitting structures other than cartilage or TFCC. ROIs were then copied to the corresponding positions on T2 and T2* maps [1]. Mean T2 and T2* values were calculated for each ROI and used for further analysis.

The imaging evaluation for T2 and T2* at 3 T and 7 T was repeated by a second radiologist (E.B.) with four years of experience in musculoskeletal MRI. This second evaluation was performed independently from the first reading for 10 randomly chosen study participants (5 controls, 5 patients) to determine inter-reader reliability. The second radiologist was blinded to the imaging evaluation of the first reading.

### Statistical analysis

Statistical analysis was performed by a biostatistician (C.G.) using R software version 4.2.2. The Wilcoxon rank-sum test was used to evaluate differences in age between patients with chronic wrist pain and healthy controls. Paired *t*-tests, supported by qq-plot check, were used to compare T2 and T2* values for different anatomic locations at 3 T or 7 T. Paired *t*-tests were also employed for the comparison of T2 and T2* values between 3 and 7 T. The use of paired *t*-tests was supported by checking the normal distribution through qq-plots for the paired differences of measurements. The correlation of T2 and T2* values between 3 and 7 T was determined by calculating Spearman’s rank correlation to account for outliers. Intraclass correlation was used to calculate the interobserver reliability. Values less than 0.50 indicate poor reliability, values between 0.50 and 0.75 indicate moderate reliability, values between 0.75 and 0.90 indicate good reliability, and values greater than 0.90 indicate excellent reliability [20]. Values of *p* lower than 0.05 were considered to indicate a statistically significant difference.

## Results

### Participant characteristics

**Fig. 3:**
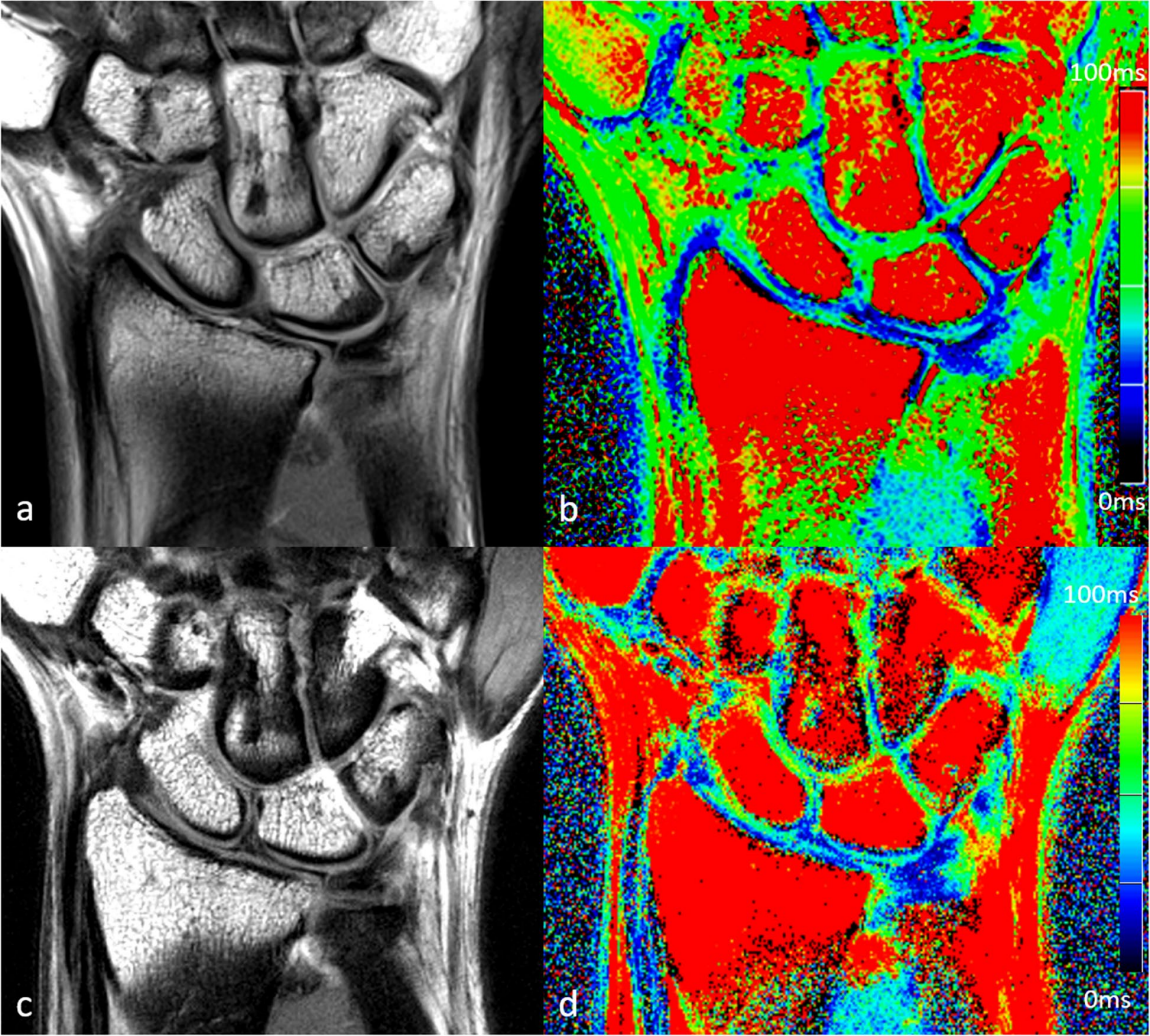
Three-Tesla (**a**, **b**) and 7-T (**c**, **d**) images of the first echo of the T2-weighted multi-echo spin-echo sequence (**a**, **c**) with corresponding color-coded maps of T2 values (**b**, **d**) of a 42-year-old patient with chronic wrist pain

**Fig. 4:**
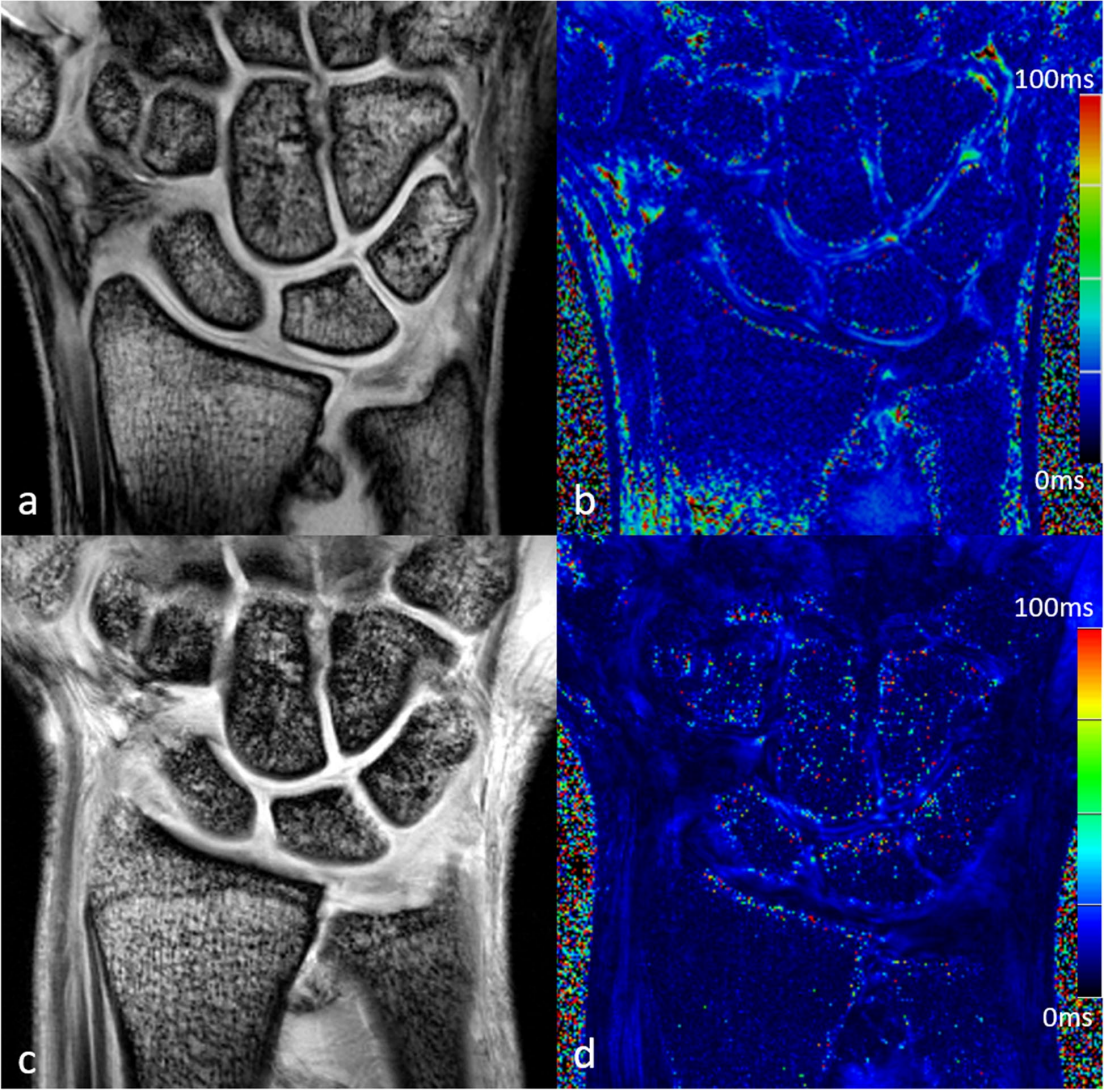
Three-Tesla (**a**, **b**) and 7-T (**c**, **d**) images of the first echo of the T2*-weighted multi-echo gradient-echo sequence (**a**, **c**) with corresponding color-coded maps of T2* values (**b**, **d**) of a 42-year-old patient with chronic wrist pain

A total of 27 healthy controls and 36 patients with chronic wrist pain were screened for study inclusion. Two healthy controls and eight patients were excluded prior to the study due to contraindications for MRI, lack of consent, or medical history. Three participants with chronic wrist pain did not complete 7-T MRI due to claustrophobia. Overall, 25 healthy controls (13 women) and 25 patients (14 men) with chronic wrist pain completed the study. The healthy controls (aged 25 ± 4 years, mean ± standard deviation) were younger than the patients with chronic wrist pain (39 ± 16 years; *p* = 0.003). Figures 3 and 4 show examples of corresponding 3-T and 7-T T2-weighted and T2*-weighted images.

### T2 and T2* values at different field strengths

At 3 T, T2 values at the cartilage of controls ranged from 32.4 ± 10.5 ms (mean ± standard deviation) at the distal radioulnar joint to 44.2 ± 7.2 at the distal lunate and proximal capitate and from 23.5 ± 5.9 ms to 29.3 ± 8.0 ms at the TFCC. T2* values at the cartilage of controls ranged from 14.2 ± 1.6 ms at the radius to 19.7 ± 3.3 ms at the distal lunate and proximal capitate and from 10.1 ± 1.2 ms to 11.3 ± 2.0 at the TFCC.

At 7 T, T2 values at the cartilage of controls ranged from 40.6 ± 15.3 ms at the distal radioulnar joint to 48.8 ± 9.8 ms at the radius and from 26.7 ± 10.7 ms to 31.7 ± 16.8 ms at the TFCC. T2* values at the cartilage of controls ranged from 8.6 ± 2.1 ms at the radius to 11.9 ± 3.0 ms at the ulnar lunate and from 6.2 ± 1.9 ms to 6.4 ± 1.4 at the TFCC. T2 and T2* values of all assessed anatomic locations and for patients with chronic wrist pain are provided in Table 1.

**Table 1:** Comparison of T2 and T2* values at 3 and 7 T for healthy controls and patients with chronic wrist pain at different anatomic locations

		Controls				Patients			
	Location	3 T	7 T	*p*-value	Correlation	3 T	7 T	*p*-value	Correlation
T2	Radius	43.8 ± 11.6	48.8 ± 9.8	0.045	0.34	38.2 ± 11.3	49.0 ± 14.1	0.002	0.45
	Scaphoid	43.9 ± 7.9	56.3 ± 15.6	0.001	0.10	46.5 ± 9.6	53.7 ± 10.2	0.01	0.03
	Lunate (radial)	34.3 ± 7.3	41.9 ± 11.0	0.03	0.05	34.7 ± 5.5	40.9 ± 9.6	0.01	0.45
	Lunate (ulnar)	34.0 ± 5.9	42.1 ± 6.4	< 0.001	0.45	35.1 ± 5.6	48.2 ± 9.4	< 0.001	0.58
	DRUJ	32.4 ± 10.5	40.6 ± 15.3	0.04	0.13	39.3 ± 18.2	45.0 ± 10.9	0.08	0.36
	Capitate/lunate	44.2 ± 7.2	46.3 ± 13.0	0.25	0.62	43.4 ± 6.2	51.5 ± 10.2	0.001	0.41
	TFCC CD	23.5 ± 5.9	26.7 ± 10.7	0.48	0.10	24.7 ± 8.5	31.2 ± 13.8	0.12	0.27
	TFCC fA	29.3 ± 8.0	31.7 ± 16.8	0.38	0.26	33.3 ± 11.2	41.2 ± 26.4	0.06	0.30
	TFCC aA	26.8 ± 6.0	29.0 ± 9.8	0.18	0.11	27.3 ± 4.3	32.9 ± 14.9	0.04	0.24
T2*	Radius	14.2 ± 1.6	8.6 ± 2.1	< 0.001	0.01	14.4 ± 2.5	8.5 ± 2.2	< 0.001	0.19
	Scaphoid	17.1 ± 3.2	10.3 ± 3.1	< 0.001	0.21	17.6 ± 3.4	10.7 ± 3.4	< 0.001	0.11
	Lunate (radial)	16.3 ± 4.3	9.4 ± 2.6	< 0.001	0.48	16.5 ± 3.3	11.1 ± 3.4	< 0.001	0.36
	Lunate (ulnar)	17.8 ± 3.1	11.9 ± 3.0	< 0.001	0.40	18.8 ± 4.4	12.5 ± 3.5	< 0.001	0.42
	DRUJ	14.4 ± 2.7	11.4 ± 3.4	0.001	0.36	17.9 ± 5.0	12.4 ± 4.3	< 0.001	0.38
	Capitate/lunate	19.7 ± 3.3	11.4 ± 3.2	< 0.001	0.13	18.2 ± 3.3	11.2 ± 3.3	< 0.001	0.01
	TFCC CD	10.1 ± 1.2	6.4 ± 1.4	< 0.001	0.28	10.9 ± 1.5	7.4 ± 2.0	< 0.001	0.41
	TFCC fA	11.3 ± 2.0	6.2 ± 1.9	< 0.001	0.04	12.7 ± 3.0	7.7 ± 2.9	< 0.001	0.03
	TFCC aA	10.5 ± 1.5	6.3 ± 2.0	< 0.001	0.24	13.2 ± 3.0	7.8 ± 2.7	< 0.001	0.47

Data are given as mean ± standard deviation; *p*-values and Spearman’s rank correlation are given for the comparison between 3 and 7 T. *aA* Apical attachment of the TFCC, *CD* Central disk of the TFCC, *fA* Foveal attachment of the TFCC, *DRUJ* Distal radioulnar joint, *TFCC* Triangular fibrocartilage complex

T2 and T2* values of the cartilage differed significantly between 3- and 7-T MRI for all anatomic locations assessed except T2 values at distal lunate and proximal capitate in controls (44.2 ± 7.2 ms *versus* 46.3 ± 13.0 ms, *p* = 0.254). At the TFCC, the only significant difference between field strengths observed for T2 values was for the apical attachment of the TFCC in patients (27.3 ± 4.3 ms *versus* 32.9 ± 14.9 ms, *p* = 0.042). T2* values differed significantly between 3 and 7 T in every anatomic location of the TFCC (Table 1).

The Spearman rank correlation between 3 and 7 T ranged from 0.03 to 0.62 for T2 time values. For T2* values, correlation coefficients between 3 and 7 T ranged from 0.01 to 0.48 (Table 1). Overall, the correlation between the field strengths was poor for both the cartilage and the TFCC.

### T2 and T2* values at different anatomic locations

The comparison of T2 and T2* values between pairs of anatomic locations (separated into cartilage and TFCC) for healthy controls is presented in Table 2. For cartilage, 9/15 comparisons at 3 T (60%; *p*-values from < 0.001 to 0.035) and 11/15 comparisons at 7 T (73%; *p*-values from < 0.001 to 0.049) showed significant differences for T2 time values. At the TFCC, T2 values showed significant differences between anatomic locations in 3/3 comparisons at 3 T (100%; *p*-values from < 0.001 to 0.018), while no differences were observed at 7 T (*p*-values from 0.060 to 0.472).

**Table 2:** Comparison of T2 and T2* values at different anatomic locations at 3 T and 7 T in healthy controls

	T2		T2*	
Location	3 T	7 T	3 T	7 T
Radius *versus* scaphoid	0.035	0.024	< 0.001	0.045
Radius *versus* lunate (radial)	0.005	0.004	0.03	0.224
Radius *versus* lunate (ulnar)	0.002	0.074	< 0.001	< 0.001
Radius *versus* DRUJ	0.102	0.01	0.74	0.001
Radius *versus* capitate/lunate	0.053	0.665	< 0.001	0.003
Scaphoid *versus* lunate (radial)	< 0.001	< 0.001	0.37	0.29
Scaphoid *versus* lunate (ulnar)	< 0.001	< 0.001	0.36	0.04
Scaphoid *versus* DRUJ	0.001	< 0.001	0.003	0.38
Scaphoid *versus* capitate/lunate	0.329	0.049	< 0.001	0.37
Lunate (radial) *versus* lunate (ulnar)	0.933	0.024	0.06	0.004
Lunate (radial) *versus* DRUJ	0.531	0.629	0.053	0.03
Lunate (radial) *versus* capitate/lunate	< 0.001	0.002	< 0.001	0.045
Lunate (ulnar) *versus* DRUJ	0.588	0.138	< 0.001	0.58
Lunate (ulnar) *versus* capitate/lunate	< 0.001	0.045	0.02	0.40
DRUJ *versus* capitate/lunate	0.001	0.023	< 0.001	0.81
TFCC CD *versus* TFCC fA	< 0.001	0.06	0.001	0.60
TFCC CD *versus* TFCC aA	0.018	0.472	0.09	0.84
TFCC fA *versus* TFCC aA	0.006	0.092	0.07	0.74

*aA* Apical attachment of the TFCC, *CD* Central disk of the TFCC, *fA* Foveal attachment of the TFCC, *DRUJ* Distal radioulnar joint, *TFCC* Triangular fibrocartilage complex

For T2* values of cartilage, significant differences were seen in 10/15 comparisons at 3 T (67%; *p*-values from < 0.001 to 0.031) and in 8/15 comparisons at 7 T (53%; *p*-values from < 0.001 to 0.045). At the TFCC, T2* values differed significantly between anatomic locations in 3/3 comparisons at 3 T (100%; *p*-values from 0.001 to 0.092). No differences were revealed for the same comparison at 7 T (*p*-values from 0.602 to 0.837).

### Inter-reader reliability

The inter-reader reliability for T2 and T2* values, measured as intraclass correlation coefficient, ranged from 0.03 to 0.98 at different anatomic locations, with most correlation coefficients (64%) being higher than 0.75, indicating good reliability. No systematic differences in reliability between 3- and 7-T MRI were observed. Supplementary Table S2 provides a detailed overview of the inter-reader reliability.

## Discussion

In our comparative study, we found that T2 and T2* values of the cartilage and T2* values of the TFCC differed significantly between 3 and 7 T. Overall, the correlation for T2 and T2* values between field strengths was poor for both cartilage and the TFCC. Most T2 and T2* values of articular cartilage varied between anatomic locations in healthy volunteers at both 3 T and 7 T. At 3 T, this was also true for the TFCC, whereas no differences were observed between anatomic locations of the TFCC at 7 T.

To our knowledge, no previous studies have assessed T2 or T2* mapping techniques of the wrist at 7-T MRI, despite potential benefits related to its inherently high contrast-to-noise and signal-to-noise ratios compared to lower field strengths [9]. Further, data at 3 T are sparse and systematic comparisons between field strengths or between anatomic locations are lacking despite these data being necessary for a valid interpretation of T2 and T2* values in patients.

Compositional MRI techniques such as T2 and T2* mapping enable noninvasive tissue quantification and thereby provide information about structural changes and tissues’ molecular status [5, 8, 21]. T2 and T2* times are affected by the orientation of collagen, collagen content, and tissue hydration and have been used in a variety of studies at different joints [22]. Studies performing compositional MRI techniques at the wrist are rare, which may be related to difficulties in image acquisition and quantification due to the small size of the anatomic structures at the wrist [1]. Götestrand et al. [12] reported a superior depiction of several anatomical structures, including the articular cartilage and different parts of the TFCC, for 7 T compared with 3 T in a small sample of healthy volunteers, emphasizing the potential of wrist imaging at ultra-high-field MRI [23]. We recently confirmed these results for cartilage imaging by comparing the image quality between 3- and 7-T MRI in a multi-reader assessment of the current study cohort. The potential superiority of image quality at 7-T MRI may be used for early disease detection of osteoarthritis in the future, as timely diagnosis and precise treatment remain essential to prevent rapid disease progression in unstable conditions of the wrist [24]. Beyond image quality, risks to patients and discomfort of ultra-high field MRI must also be considered. There is currently no evidence for serious health effects from acute exposure up to 8 T, but it is worth noting that patients undergoing ultra-high field MRI may experience certain discomforts and sensations due to the varying magnetic fields within the MRI scanner [25]. Some reported effects include vertigo, peripheral nerve stimulation, headache, the appearance of phosphenes, thermal heat sensation, dizziness, and unsteady gait after scanning [26–29].

Our observed differences in T2 and T2* values between 3 and 7 T support the findings of previously published studies. Although T2 and T2* relaxation times typically decrease with increasing field strength, we observed an increase in T2 relaxation times at higher magnetic field strength [30]. A similar increase was also observed by Welsch et al. in the deep layer of knee cartilage tissue [31]. One possible explanation for this unexpected increase is the fact that the magnetization decays non-exponentially (roughly biexponentially) and that at 3 T the short T2 component still contributes to the signal, whereas at 7 T the short component is probably too short and the longer components dominate the signal. Similar effects might occur in wrist cartilage. However, differences between 3 and 7 T in our study might be related to not only different field strengths, but also the different designs of the wrist coils used. In particular, the applied transmit field (B_1_^+^) has an influence on the measured T2 relaxation times [32]. B_1_^+^ is typically more inhomogeneous at 7 T than at 3 T, which might explain the larger standard deviations of the measured T2 relaxation times [30]. In a previous study evaluating 15 volunteers at 3 T, it was observed that different coils caused significant alterations in T2 and T2* values of the cartilage at the patella [33]. In addition, Chang et al. [34] reported variations in T2 values at the femoral and tibial cartilage when comparing a 28-channel receive array coil and a quadrature volume coil at 7 T.

Beyond differences in field strengths, coil design and pulse sequences causing alterations in T2 and T2* values intra-individual variations between different anatomic locations must also be considered [33, 35, 36]. Differences between anatomic locations, even within the same joint, may be related to physiological variations, functional demand, and compression load [37–39]. Local variations in T2 and T2* values of the cartilage of the knee at 7 T were described in healthy volunteers in 2008 [18]. Subburaj et al. [17] also reported regional variations in compositional MRI including T2 mapping of hip joint cartilage in healthy controls and patients with femoro-acetabular impingement using a 3-T scanner. The magic angle effect must be also considered as another relevant factor causing variations of compositional imaging between different anatomic locations [40]. *In vivo* and *ex vivo* studies have shown a strong magic angle effect on T2 values of cartilage with changes of more than 200%, especially in the deeper layers of cartilage [40, 41]. Our findings at the wrist support the findings of these previous studies, as we observed significant differences in T2 and T2* values for articular cartilage between different anatomic locations at 3 T and 7 T. Differences between the measured T2 values at the scaphoid and at the lunatum in our study may be a consequence of the magic angle effect.

However, at the TFCC, significant regional differences occurred at 3 T, but not at 7 T, for both T2 and T2*. The variation in T2 and T2* values between different anatomic locations in healthy controls suggests that single regional values must be interpreted cautiously. It also emphasizes the need to implement local reference values for each anatomic location, which need to be ascertained on the deployed hardware using the respective sequence protocol [6]. Due to the many factors affecting T2 and T2* values, intra-individual comparison using baseline and follow-up examinations acquired in an identical fashion on the same MR system may currently be the most feasible clinical approach to obtain consistent and reliable results without the enormous burden of establishing local reference values. We did not find differences in inter-reader reliability between 3 and 7 T. Considering only these reliability data, our results do not suggest a definite advantage of performing T2 and T2* mapping at the wrist at ultra-high-field MRI. However, our results show the difficulties of the assessment of reliable T2 and T2* values of small anatomic structures, as correlation coefficients ranged from 0.03 to 0.98 with around one third (36%) of correlation coefficients being smaller than 0.75.

Like any quantitative imaging technique, compositional MRI of cartilage requires careful attention to detail across the acquisition and analysis pipeline to ensure that values obtained are accurate, reproducible, and interpretable. Previous studies have shown marked variability across vendors, radiofrequency coils, and pulse sequences [35, 42–45]. To address some of these challenges, a musculoskeletal subcommittee was formed under the Radiology Society of North America (RSNA) Quantitative Imaging Biomarkers Alliance (QIBA) task force in 2017. The first profile of the committee included recommendations aimed at standardizing data acquisition techniques for cartilage T2 and T1ρ imaging as potential imaging biomarkers [46]. However, though compositional MRI techniques have been available for more than 20 years, they have not yet made a real impact on clinical care. This may be due in part to some of the technical challenges described above, such as standardization [47]. However, another major reason is the lack of disease-modifying drug treatments for cartilage restoration and osteoarthritis, which means that the identification of early disease by compositional MRI currently has no or only limited impact on patient management [47]. This is likely to change in the future once disease-modifying approaches are available that may require longitudinal monitoring of structural treatment effects.

Our study has some limitations: first, the small number of study participants with missing reference standard providing a final diagnosis in the case of the included patients. Further, the uniqueness of the study population limits the generalization of our data and the transferability to other wrist conditions. Second, due to ethical considerations, we cannot provide any histological correlation that objectively validates the MRI findings. Third, the sequences we used for 7-T MRI were optimized and adapted from standard sequences used at 3 T so that measurement times were comparable between field strengths. For T2* mapping at 7 T, we had to use shorter echo times due to the increased susceptibility artifacts at longer echo times. This might also have affected the results. Fourth, the coil design geometry used was different for 3 T and 7 T; thus, the wrist position was slightly different during image acquisition. Moreover, at 7 T, B_1_^+^ inhomogeneities might have contributed to the variation in measured T2 times.

In conclusion, T2 and T2* mapping are feasible for compositional imaging of the TFCC and the cartilage at the wrist at both 3 T and 7 T. As observed in other joints, quantitative T2 and T2* time values differ between field strengths. Further, T2 and T2* times vary between anatomic locations and do not show a strong correlation between 3 and 7 T, which may be of high importance for valid imaging interpretation and emphasizes the need to establish reference values for healthy individuals for every anatomic location.

### Supplementary Information

**Additional file 1: Table S1. Table S2. **

## Data Availability

The datasets used and/or analyzed during the current study are available from the corresponding author upon reasonable request.

## Abbreviations

MRI: Magnetic resonance imaging
ROI: Region of interest
TE: Echo time
TFCC: Triangular fibrocartilage complex
TR: Repetition time

## References

[1] Rehnitz C, Klaan B, Burkholder I, von Stillfried F, Kauczor HU, Weber MA (2017). Delayed gadolinium-enhanced MRI of cartilage (dGEMRIC) and T(2) mapping at 3T MRI of the wrist: Feasibility and clinical application. J Magn Reson Imaging.

[2] Hayter CL, Gold SL, Potter HG (2013). Magnetic resonance imaging of the wrist: bone and cartilage injury. J Magn Reson Imaging.

[3] Nagy L (2005). Salvage of post-traumatic arthritis following distal radius fracture. Hand Clin.

[4] Rettig AC (2003). Athletic injuries of the wrist and hand. Part I: traumatic injuries of the wrist. Am J Sports Med.

[5] Hesper T, Hosalkar HS, Bittersohl D (2014). T2*mapping for articular cartilage assessment: principles, current applications, and future prospects. Skeletal Radiol.

[6] Heiss R, Janka R, Uder M, Nagel AM, Trattnig S, Roemer FW (2019). Update cartilage imaging of the small joints Focus on high-field MRI. Radiologe.

[7] Crema MD, Roemer FW, Marra MD (2011). Articular cartilage in the knee: current MR imaging techniques and applications in clinical practice and research. Radiographics.

[8] Newbould RD, Miller SR, Toms LD (2012). T2* measurement of the knee articular cartilage in osteoarthritis at 3T. J Magn Reson Imaging.

[9] Krug R, Stehling C, Kelley DA, Majumdar S, Link TM (2009). Imaging of the musculoskeletal system in vivo using ultra-high field magnetic resonance at 7 T. Invest Radiol.

[10] Rauscher I, Bender B, Grozinger G (2014). Assessment of T1, T1rho, and T2 values of the ulnocarpal disc in healthy subjects at 3 tesla. Magn Reson Imaging.

[11] Yan M, Wen S, Wang X (2022) Quantitative analysis of triangular fibrocartilage complex injury by 3.0T MR 3D VIBE and T2 mapping techniques. Medicine (Baltimore) 101:e31589. 10.1097/MD.0000000000031589 10.1097/MD.0000000000031589 PMC9794244 36595773

[12] Heiss R, Weber MA, Balbach E et al (2023) Clinical application of ultrahigh-field-strength wrist MRI: a multireader 3-T and 7-T comparison study. Radiology. 10.1148/radiol.220753 10.1148/radiol.220753 36625744

[13] Donati OF, Nordmeyer-Massner J, Nanz D (2011). Direct MR arthrography of cadaveric wrists: comparison between MR imaging at 3.0T and 7.0T and gross pathologic inspection. J Magn Reson Imaging.

[14] Friedrich KM, Komorowski A, Trattnig S (2012). 7T imaging of the wrist. Semin Musculoskelet Radiol.

[15] Nordmeyer-Massner JA, Wyss M, Andreisek G, Pruessmann KP, Hodler J (2011). In vitro and in vivo comparison of wrist MR imaging at 3.0 and 7.0 tesla using a gradient echo sequence and identical eight-channel coil array designs. J Magn Reson Imaging.

[16] Raval SB, Zhao T, Krishnamurthy N (2016). Ultra-high-field RF coil development for evaluating upper extremity imaging applications. NMR Biomed.

[17] Subburaj K, Valentinitsch A, Dillon AB (2013). Regional variations in MR relaxation of hip joint cartilage in subjects with and without femoralacetabular impingement. Magn Reson Imaging.

[18] Welsch GH, Mamisch TC, Hughes T (2008). In vivo biochemical 7.0 Tesla magnetic resonance: preliminary results of dGEMRIC, zonal T2, and T2* mapping of articular cartilage. Invest Radiol.

[19] Bae WC, Ruangchaijatuporn T, Chang EY (2016). MR morphology of triangular fibrocartilage complex: correlation with quantitative MR and biomechanical properties. Skeletal Radiol.

[20] Koo TK, Li MY (2016). A guideline of selecting and reporting intraclass correlation coefficients for reliability research. J Chiropr Med.

[21] Mosher TJ, Dardzinski BJ (2004). Cartilage MRI T2 relaxation time mapping: overview and applications. Semin Musculoskelet Radiol.

[22] Emanuel KS, Kellner LJ, Peters MJM, Haartmans MJJ, Hooijmans MT, Emans PJ (2022). The relation between the biochemical composition of knee articular cartilage and quantitative MRI: a systematic review and meta-analysis. Osteoarthritis Cartilage.

[23] Gotestrand S, Bjorkman A, Bjorkman-Burtscher IM (2022). Visualization of wrist anatomy-a comparison between 7T and 3T MRI. Eur Radiol.

[24] Grunz JP, Gietzen CH, Christopoulos G (2021). Osteoarthritis of the wrist: pathology, radiology, and treatment. Semin Musculoskelet Radiol.

[25] International Commission on Non-Ionizing Radiation P (2009). Guidelines on limits of exposure to static magnetic fields. Health Phys.

[26] Theysohn JM, Maderwald S, Kraff O, Moenninghoff C, Ladd ME, Ladd SC (2008). Subjective acceptance of 7 Tesla MRI for human imaging. MAGMA.

[27] Hansson B, Hoglund P, Markenroth Bloch K (2019). Short-term effects experienced during examinations in an actively shielded 7 T MR. Bioelectromagnetics.

[28] Hansson B, Markenroth Bloch K, Owman T (2020). Subjectively reported effects experienced in an actively shielded 7T MRI: a large-scale study. J Magn Reson Imaging.

[29] Friebe B, Wollrab A, Thormann M (2015). Sensory perceptions of individuals exposed to the static field of a 7T MRI: a controlled blinded study. J Magn Reson Imaging.

[30] Ladd ME, Bachert P, Meyerspeer M (2018). Pros and cons of ultra-high-field MRI/MRS for human application. Prog Nucl Magn Reson Spectrosc.

[31] Welsch GH, Apprich S, Zbyn S (2011). Biochemical (T2, T2* and magnetisation transfer ratio) MRI of knee cartilage: feasibility at ultra-high field (7T) compared with high field (3T) strength. Eur Radiol.

[32] Agt RP, Neji R, Wood TC, Baburamani AA, Malik SJ, Hajnal JV (2020). Controlled saturation magnetization transfer for reproducible multivendor variable flip angle T(1) and T(2) mapping. Magn Reson Med.

[33] Pachowsky ML, Trattnig S, Apprich S, Mauerer A, Zbyn S, Welsch GH (2013). Impact of different coils on biochemical T2 and T2* relaxation time mapping of articular patella cartilage. Skeletal Radiol.

[34] Chang G, Wiggins GC, Xia D (2012). Comparison of a 28-channel receive array coil and quadrature volume coil for morphologic imaging and T2 mapping of knee cartilage at 7T. J Magn Reson Imaging.

[35] Matzat SJ, McWalter EJ, Kogan F, Chen W, Gold GE (2015). T2 Relaxation time quantitation differs between pulse sequences in articular cartilage. J Magn Reson Imaging.

[36] McKenzie CA, Williams A, Prasad PV, Burstein D (2006). Three-dimensional delayed gadolinium-enhanced MRI of cartilage (dGEMRIC) at 1.5T and 3.0T. J Magn Reson Imaging.

[37] Shepherd DE, Seedhom BB (1999). Thickness of human articular cartilage in joints of the lower limb. Ann Rheum Dis.

[38] Mosher TJ, Dardzinski BJ, Smith MB (2000). Human articular cartilage: influence of aging and early symptomatic degeneration on the spatial variation of T2–preliminary findings at 3 T. Radiology.

[39] Nishii T, Tanaka H, Sugano N, Sakai T, Hananouchi T, Yoshikawa H (2008). Evaluation of cartilage matrix disorders by T2 relaxation time in patients with hip dysplasia. Osteoarthritis Cartilage.

[40] Shao H, Pauli C, Li S (2017). Magic angle effect plays a major role in both T1rho and T2 relaxation in articular cartilage. Osteoarthritis Cartilage.

[41] Mosher TJ, Smith H, Dardzinski BJ, Schmithorst VJ, Smith MB (2001). MR imaging and T2 mapping of femoral cartilage: in vivo determination of the magic angle effect. AJR Am J Roentgenol.

[42] Balamoody S, Williams TG, Wolstenholme C (2013). Magnetic resonance transverse relaxation time T2 of knee cartilage in osteoarthritis at 3-T: a cross-sectional multicentre, multivendor reproducibility study. Skeletal Radiol.

[43] Dardzinski BJ, Schneider E (2013). Radiofrequency (RF) coil impacts the value and reproducibility of cartilage spin-spin (T2) relaxation time measurements. Osteoarthritis Cartilage.

[44] Kim J, Mamoto K, Lartey R (2020). Multi-vendor multi-site T(1rho) and T(2) quantification of knee cartilage. Osteoarthritis Cartilage.

[45] Pai A, Li X, Majumdar S (2008). A comparative study at 3 T of sequence dependence of T2 quantitation in the knee. Magn Reson Imaging.

[46] Chalian M, Li X, Guermazi A (2021). The QIBA Profile for MRI-based Compositional Imaging of Knee Cartilage. Radiology.

[47] Hayashi D, Roemer FW, Tol JL (2023). Emerging quantitative imaging techniques in sports medicine. Radiology.

