# Correction: Variation in cartilage T2 and T2* mapping of the wrist: a comparison between 3- and 7-T MRI

**DOI:** 10.1186/s41747-024-00449-x

**Published:** 2024-03-16

**Authors:** Rafael Heiss, Marc-André Weber, Eva L. Balbach, Maximilian Hinsen, Frederik Geissler, Armin M. Nagel, Mark E. Ladd, Andreas Arkudas, Raymund E. Horch, Christine Gall, Michael Uder, Frank W. Roemer

**Affiliations:** 1 Department of Radiology, University Hospital Erlangen, Friedrich-Alexander-Universität Erlangen-Nürnberg (FAU), Maximiliansplatz 3, 91054 Erlangen, Germany; 2 Institute of Diagnostic and Interventional Radiology, Pediatric Radiology and Neuroradiology, University Medical Center Rostock, Schillingallee 35, 18057 Rostock, Germany; 3 Medical Physics in Radiology, German Cancer Research Center (DKFZ), Im Neuenheimer Feld 280, 69120 Heidelberg, Germany; 4 Faculty of Medicine and Faculty of Physics and Astronomy, Heidelberg University, Im Neuenheimer Feld 226, 69120 Heidelberg, Germany; 5 Department of Plastic and Hand Surgery and Laboratory for Tissue Engineering and Regenerative Medicine, University Hospital Erlange, Friedrich-Alexander-Universität Erlangen-Nürnberg (FAU), Krankenhausstraße 12, 91054 Erlangen, Germany; 6 Institute for Medical Informatics, Biometry and Epidemiology, Friedrich-Alexander-Universität Erlangen-Nürnberg (FAU), Waldstraße 6, 91054 Erlangen, Germany; 7 Boston University School of Medicine, 72 E Concord St, Boston, MA 02118 USA

**Correction** **:** **Eur Radiol Exp 7, 80 (2023)**

**https://doi.org/10.1186/s41747-023-00394-1**

The original article [1] omits the following funding information that the authors would like to note in this correction article:

“We acknowledge financial support by Deutsche Forschungsgemeinschaft and Friedrich-Alexander-Universität Erlangen-Nürnberg within the funding programme "Open Access Publication Funding".”
