# Supplementary material for: Variation in cartilage T2 and T2* mapping of the wrist: a comparison between 3- and 7-T MRI
Source: Eur Radiol Exp. 2023 Dec 14;7:80. doi: 10.1186/s41747-023-00394-1 (PMC10719234; doi:10.1186/s41747-023-00394-1)
Supplement: Supplementary file 1 — Additional file 1: Table S1. Table S2. [file 41747_2023_394_MOESM1_ESM.docx]

**Variation in cartilage T2 and T2* mapping of the wrist: a comparison between 3-T and 7-T MRI**

**ELECTRONIC SUPPLEMENTARY MATERIAL**

| **Parameter** | **3T** | | **7T** | |
| --- | --- | --- | --- | --- |
|  | **T2 mapping** | **T2* mapping** | **T2 mapping** | **T2* mapping** |
| **Plane of Acquisition** | coronal | coronal | coronal | coronal |
| **Fat Suppression** | No | No | No | No |
| **Repetition Time (ms)*** | 2000 | 648 | 2000 | 648 |
| **Echo Time (ms)** | 16.1, 32.2, 48.3, 64.4, 80.5 | 6.04, 16.94, 27.84, 38.74, 49.64 | 16.1, 32.2, 48.3, 64.4, 80.5 | 4.08, 7.01, 9.62, 12.23, 15.29 |
| **Acquisition Time (min:sec)** | 06:52 | 04:10 | 6:52 | 4:02 |
| **Voxel Size (mm)** | 0.3 x 0.3 x 3 | 0.3 x 0.3 x 3 | 0.3 x 0.3 x 2 | 0.3 x 0.3 x 3 |
| **Averages** | 1 | 1 | 1 | 1 |
| **Concatenations** | 1 | 1 | 1 | 1 |
| **Slices** | 10 | 10 | 10 | 10 |
| **Distance Factor (%)** | 10 | 10 | 10 | 100 |
| **Phase Oversampling (%)** | 0 | 0 | 0 | 0 |
| **FoV Read (mm)** | 100 | 100 | 100 | 100 |
| **FoV Phase (%)** | 100 | 100 | 100 | 100 |
| **Base Resolution** | 384 | 384 | 384 | 384 |
| **Phase Resolution (%)** | 100 | 100 | 100 | 100 |
| **Flip Angle (°)** | 180 | 60 | 180 | 60 |
| **Interpolation** | Off | Off | Off | Off |
| **Phase Encoding Direction** | RL | RL | RL | RL |
| **Phase Partial Fourier** | 4/8 | Off | 4/8 | Off |
| **Echo Spacing (ms)** | n/a | n/a | n/a | n/a |
| **Turbo Factor (echo train length)** | n/a | n/a | n/a | n/a |
| **Echo Trains per Slice** | n/a | n/a | n/a | n/a |
| **Receiver Bandwidth (Hz/pixel)** | 228 | 260 | 434 | 470 |
| **Readout Duty Cycle (%)** | n/a | n/a | n/a | n/a |
| **Acceleration Technique** | No | No | No | No |
| **Acceleration Factor** | n/a | n/a | n/a | n/a |

**Table S1:** Detailed MRI parameters of acquired sequences. ms, milliseconds; min, minutes; sec, seconds; mm, millimeter; RL, right–left; n/a, not applicable.

|  |  | **Controls** | | **Patients** | |
| --- | --- | --- | --- | --- | --- |
|  |  | **3T** | **7T** | **3T** | **7T** |
| **T2** | **Radius** | 0.63 | 0.95 | 0.97 | 0.98 |
|  | **Scaphoid** | 0.63 | 0.73 | 0.03 | 0.62 |
|  | **Lunate (radial)** | 0.88 | 0.36 | 0.84 | 0.78 |
|  | **Lunate (ulnar)** | 0.14 | 0.88 | 0.72 | 0.92 |
|  | **DRUJ** | 0.88 | 0.83 | 0.93 | 0.76 |
|  | **Capitate/Lunate** | 0.95 | 0.59 | 0.96 | 0.96 |
|  | **TFCC CD** | 0.89 | 0.64 | 0.67 | 0.32 |
|  | **TFCC fA** | 0.70 | 0.80 | 0.48 | 0.67 |
|  | **TFCC aA** | 0.74 | 0.85 | 0.03 | 0.46 |
| **T2*** | **Radius** | 0.80 | 0.79 | 0.97 | 0.95 |
|  | **Scaphoid** | 0.96 | 0.93 | 0.85 | 0.73 |
|  | **Lunate (radial)** | 0.69 | 0.44 | 0.69 | 0.36 |
|  | **Lunate (ulnar)** | 0.80 | 0.93 | 0.85 | 0.88 |
|  | **DRUJ** | 0.95 | 0.88 | 0.92 | 0.83 |
|  | **Capitate/Lunate** | 0.98 | 0.99 | 0.96 | 0.59 |
|  | **TFCC CD** | 0.90 | 0.99 | 0.05 | 0.64 |
|  | **TFCC fA** | 0.77 | 0.97 | 0.45 | 0.80 |
|  | **TFCC aA** | 0.98 | 0.37 | 0.18 | 0.85 |

**Table S2:** The inter-reader agreement for T2 and T2* values presented as the intraclass correlation coefficient for two readers at each anatomic location. DRUJ, distal radioulnar joint; TFCC, triangular fibrocartilage complex; CD, central disc of the TFCC; fA, foveal attachment of the TFCC; aA, apical attachment of the TFCC.
